# MRI Findings of Causalgia of the Lower Extremity Following Transsphenoidal Resection of Pituitary Tumor

**DOI:** 10.1155/2012/598048

**Published:** 2012-09-13

**Authors:** D. Ryan Ormond, Augustine L. Moscatello, Raj Murali

**Affiliations:** ^1^Department of Neurosurgery, New York Medical College, Munger Pavilion, Third Floor, Valhalla, NY 10595, USA; ^2^Department of Otolaryngology, New York Medical College, Valhalla, NY 10595, USA

## Abstract

*Background*. Causalgia is continuing pain, allodynia, or hyperalgesia after nerve injury with edema, changes in skin blood flow, or abnormal sudomotor activity. Here we report a case of lower extremity causalgia following elective transsphenoidal resection of a pituitary tumor in a young man. *Clinical Presentation*. A 33-year-old man with acromegaly underwent elective sublabial transsphenoidal resection of his pituitary tumor. During the three-hour surgery, the lower limbs were kept in a supine, neutral position with a pillow under the knees. The right thigh was slightly internally rotated with a tape to expose fascia lata, which was harvested to repair the sella. Postoperatively, he developed causalgia in a distal sciatic and common peroneal nerve distribution. Pain was refractory to several interventions. Finally, phenoxybenzamine improved his pain significantly. *Conclusions*. Malpositioning in the operating room resulted in causalgia in this young man. Phenoxybenzamine improved, and ultimately resolved, his symptoms. Improvement in his pain symptoms correlated with resolution of imaging changes in the distal sciatic and peroneal nerves on the side of injury.

## 1. Introduction

Complex regional pain syndrome Type II (causalgia) is defined as the presence of continuing pain, allodynia, or hyperalgesia after a nerve injury with evidence of edema, changes in skin blood flow, or abnormal sudomotor activity near the area of pain [1]. It is a diagnosis of exclusion when no other explanation is found after extensive workup. It differs from the complex regional pain syndrome Type I (reflex sympathetic dystrophy) in that it occurs often in a regional distribution in the body rather than in a more crisp peripheral nerve distribution [2]. Causalgia, too, can extend beyond a peripheral nerve distribution, but this is typically later in the course of disease [3].

Causalgia was first described by Denmark in 1813, with the term “causalgia” coined by Silas Weir Mitchell in 1864 [4]. It is most common after traumatic partial injury of a nerve and causes neuropathic pain that is often refractory to traditional analgesics. It is believed to be caused by aberrant firing of sympathetic neurons. A review of the literature in 2003 found a much higher reported incidence during times of war (877 reported cases, or 65%) [4]. The vast majority of cases reported in the literature occur following high-velocity trauma (76.7% of cases with a known etiology). Only 36 of 1538 cases occurred after surgery and were most often due to direct nerve injury during the procedure [4]. The vast majority (all but 93 patients) had symptom onset within one month of injury. Symptoms commonly include burning pain, increased sweating, cold sensitivity, warmness or cyanosis of extremity, paresthesias, dysesthesias, or allodynia.

Treatment of causalgia has been diverse, ranging from medical to surgical in nature. This simply reflects the refractory nature of the pain to treatment. Medical agents of neuropathic pain have often been used with mixed results [2, 3]. Surgical management has included sympathetic chain anesthetic blocks or surgical sympathectomy [4]. Many authors advocate sympathectomy as the “gold standard” of treatment of this condition, but results are mixed at best, with potential significant complications including worsening pain, a new pain syndrome, or abnormal forms of sweating [5, 6]. Cochrane review of both sympathetic block and sympathectomy concludes that there is insufficient evidence to advocate either practice [5, 6]. More recently, spinal cord stimulation has also been attempted to control causalgia pain with mixed results [1, 7].

Here we report a case of right distal sciatic and common peroneal nerve causalgia following elective transsphenoidal resection of a growth hormone secreting pituitary tumor in a young man.

## 2. Case Report

### 2.1. HPI

A 33-year-old RH male presented to his primary medical doctor with the complaint of severe, unremitting headaches for four years. He also had hand, foot, and jaw growth over this same time period. He denied any nausea or vomiting, weakness, numbness, or changes in his vision. He denied any toxic habits.

### 2.2. Physical Examination

**Figure 1:**
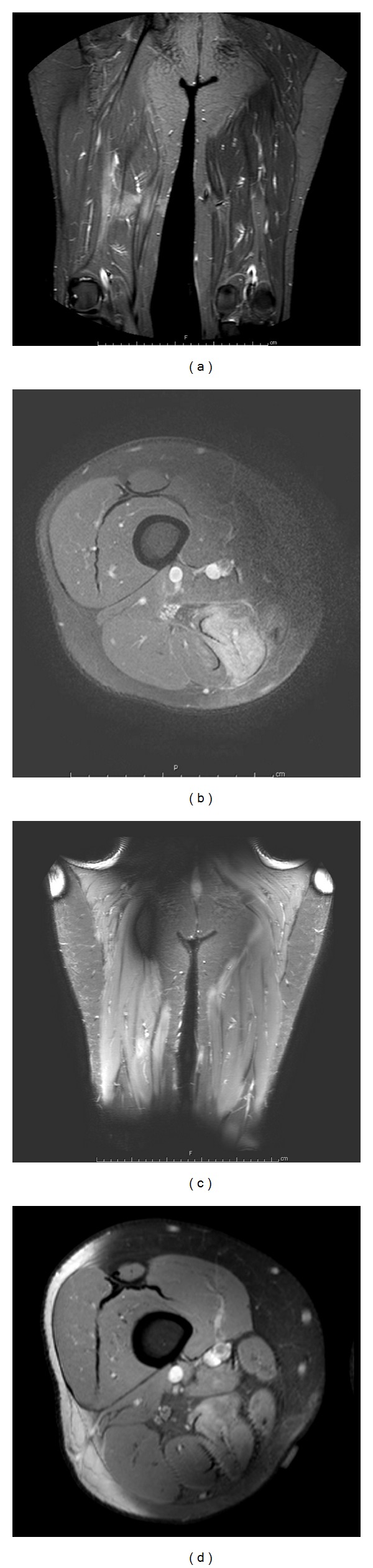
(a) and (b) Initial MRI of the lower extremities demonstrates edema in the right semimembranosis and semitendinosis muscles with abnormal enhancement in the distal sciatic and common peroneal nerves. (c) and (d) Delayed MRI of the lower extremities demonstrates near resolution of edema in the muscle and normalization of the nerves without abnormal enhancement.

The patient was awake, alert, and oriented. He had macroglossia, with significant acromegalic facies, and large hands and feet. He was mildly hypertensive. His neurological examination was grossly normal. MR imaging demonstrated a pituitary microadenoma. His insulin-like growth factor 1 (IGF-1) level was 747 ng/mL. He underwent elective sublabial transsphenoidal resection of pituitary tumor. During the three-hour surgery, the lower limbs were kept in a supine, neutral position with a pillow under the knees. The right thigh was slightly internally rotated with a tape to expose fascia lata, which was harvested to repair the sella. Postoperatively, he had rapid normalization of his IGF-1 levels, and his headaches went away. However, immediately upon waking in the operating room, he complained of pain in his right lower extremity that was burning and aching in nature. This quickly progressed to allodynia, and the patient no longer could walk due to pain. Pain was in a distal sciatic and common peroneal nerve distribution. Pain was refractory to opioids, topical analgesics, or traditional agents for neuropathic pain like gabapentin, pregabalin, and carbamazepine. MRI of the lower extremities demonstrated edema in the semimembranosis and semitendinosis muscles with abnormal enhancement of the distal sciatic and common peroneal nerves on the right side (Figures 1(a) and 1(b)). Nerve conduction studies showed a sensory neuropathy in a sciatic nerve distribution. Medications attempted with no improvement in his symptoms included gabapentin, pregabalin, carbamazepine, oxcarbazapine, opioids, clonidine, baclofen, various NSAIDS, and topical anesthetics [8]. Sympathetic nerve block gave some transient, mild relief of his symptoms. Finally, slowly escalating doses of phenoxybenzamine, a long-acting noncompetitive alpha-adrenergic inhibitor of postganglionic synapses, resulted in improvement and ultimate resolution of his symptoms. Follow-up MRI after near-resolution of symptoms demonstrated decreased edema in the right semimembranosis and semitendinosis muscles and normal appearance of the distal sciatic and common peroneal nerves (Figures 1(c) and 1(d)).

## 3. Discussion

Causalgia is an extremely rare iatrogenic complication of surgery, representing only 36 of 1538 reported cases [4]. This acromegalic patient awoke from surgery with increasing symptoms of causalgia in a distal sciatic and common peroneal nerve distribution over the course of a few weeks. This pain was constant and refractory to many medical therapies. Finally, slowly escalating oral doses of phenoxybenzamine, a long-acting noncompetitive alpha-adrenergic inhibitor of postganglionic synapses, improved, and ultimately resolved, his causalgia pain. He was tapered off the phenoxybenzamine over the course of 4–6 weeks. He experienced minimal symptoms of orthostatic hypotension during his dose escalation.

The use of phenoxybenzamine for CRPS Type II was first reported in a retrospective series of 40 consecutive cases of causalgia occurring after shrapnel injury during war [9]. The drug was given orally beginning at 10 mg/day. The dose was gradually escalated to a total daily dose of 40 to 120 mg/day and then tapered as tolerated based on pain over 6 to 8 weeks. Total pain resolution was reported in all cases. Follow-up period ranged from 6 months to 6 years. Side effects included orthostatic hypotension and ejaculatory problems during treatment [9]. More recent case reports show some efficacy with the use of phenoxybenzamine for nonmilitary causes of causalgia [10]. Treatment early in the course of disease appears to improve efficacy of available treatments [9, 10]. Our patient underwent a battery of medication regimens and sympathetic chain blockade in an attempt to improve his symptoms of causalgia. Pain was refractory to all therapies until the use of phenoxybenzamine. Improvement in his symptoms began at a dose of 30–40 mg/day and resolved at a dose of 60 mg/day. Improvement in his pain symptoms correlated with resolution of imaging changes in the distal sciatic and peroneal nerves on the side of injury (Figures 1(a)–1(d)).

## 4. Conclusion

Malpositioning in the operating room in the setting of acromegaly likely resulted in partial nerve injury to the distal sciatic and common peroneal nerves causing causalgia in this young man. Phenoxybenzamine resulted in significant improvement and ultimate resolution of his symptoms.
